# Prediction of Auditory Performance in Cochlear Implants Using Machine Learning Methods: A Systematic Review

**DOI:** 10.3390/audiolres15030056

**Published:** 2025-05-08

**Authors:** Beyza Demirtaş Yılmaz

**Affiliations:** Department of Audiology, Faculty of Health Sciences, Erciyes University, Kayseri 38039, Turkey; beyzademirtas@erciyes.edu.tr; Tel.: +90-541-714-7207

**Keywords:** machine learning, cochlear implants, audiology, hearing loss, speech perception, speech in noise, cochlear implantation candidates

## Abstract

**Background/Objectives:** Cochlear implantation is an advantageous procedure for individuals with severe to profound hearing loss in many aspects related to auditory performance, social communication and quality of life. As machine learning applications have been used in the field of Otorhinolaryngology and Audiology in recent years, signal processing, speech perception and personalised optimisation of cochlear implantation are discussed. **Methods:** A comprehensive literature review was conducted in accordance with the PRISMA guidelines. PubMed, Scopus, Web of Science, Google Scholar and IEEE databases were searched for studies published between 2010 and 2025. We analyzed 59 articles that met the inclusion criteria. Rayyan AI software was used to classify the studies so that the risk of bias was reduced. Study design, machine learning algorithms, and audiological measurements were evaluated in the data analysis. **Results:** Machine learning applications were classified as preoperative evaluation, speech perception, and speech understanding in noise and other studies. The success rates of the articles are presented together with the number of articles changing over the years. It was observed that Random Forest, Decision Trees (96%), Bayesian Linear Regression (96.2%) and Extreme machine learning (99%) algorithms reached high accuracy rates. **Conclusions:** In cochlear implantation applications in the field of audiology, it has been observed that studies have been carried out with a variable number of people and data sets in different subfields. In machine learning applications, it is seen that a high amount of data, data diversity and long training times contribute to achieving high performance. However, more research is needed on deep learning applications in complex problems such as comprehension in noise that require time series processing. **Funding and other resources:** This study was not funded by any institution or organization. No registration was performed for this study.

## 1. Introduction

According to the World Health Organization’s 2021 report, by 2050, approximately 2.5 billion people will have some degree of hearing loss, and more than 700 million people will need rehabilitation services [1]. With advancing technology, developing programming strategies, and surgical techniques, cochlear implants are used as a safe and effective rehabilitation method [2]. Despite the increasing success of cochlear implants in recent years, there may be differences in the performance and satisfaction of individuals with hearing loss. While some individuals may experience significant improvements in hearing performance, others may experience limited improvements in performance [3,4,5]. There are various studies investigating these differences in the literature [6,7,8]. However, identifying and interpreting these predictive factors is difficult even today [4]. This shows the necessity and importance of individualized rehabilitation programs. With the changing world and developing technology, machine learning techniques have been applied in recent years to predict performance differences after cochlear implantation and to develop individualized rehabilitation programs [9,10,11,12,13,14].

The ability of computer systems to imitate human-like thinking, learning, problem-solving and decision-making is the foundation of artificial intelligence. Thanks to algorithms that can identify patterns and make predictions by extracting meaning from data, artificial intelligence gives more comprehensive results day by day. Artificial intelligence, which has taken its place in many fields such as health, education, finance and trade, continues to bring innovations in various fields. Machine learning, which is a functional branch of this broad framework, has come to the fore as a sub-field of automatic learning in order to develop artificial intelligence human-like thinking, learning and decision-making processes [15,16,17].

These algorithms are based on the principle that computer software programs can analyze data from data sets by training the program rather than coding it with specific formulas [18]. Machine learning generally enables data prediction and analysis through two classifications. In one of these classifications, supervised learning, algorithms learn the relationships between the input data and the desired output and make predictions based on new data. In unsupervised machine learning, the algorithm tries to learn the patterns and patterns in the data by making a particular classification or grouping [19,20]. Deep learning, a more advanced branch of machine learning recently used in many fields, is applied to obtain more meaningful results from large and complex data sets. Deep learning, consisting of artificial neural networks, can learn more abstract and complex situations due to its multi-stage nature.

Although the foundations of machine learning date back to earlier years in the field of engineering, it is possible to say that there have been developments in the field of Otolaryngology and Audiology in the last two decades. Since the early 2000s, it has been seen that more basic algorithms have been used in the fields of diagnostics, imaging, digital signal processing and automatic diagnostic methods in Otolaryngology and Audiology. In the development of ECAP measurements, machine learning algorithms have been used to perform automatic analysis in cochlear implantation [21,22,23,24,25].

Machine learning algorithms for predicting hearing and speech performance of individuals with hearing loss, optimization studies in cochlear implant applications, and determination of electrode placement have been developed and used since the early 2010s [10,26,27,28,29,30,31,32].

Deep learning-based solutions, especially in the field of Ear, Nose and Throat and Hearing Science, with developing algorithms, show promising results in auditory signal processing, improvement of speech intelligibility and optimization of postoperative cochlear implant planning [18,33,34,35]. Machine learning algorithms are used to predict post-implantation auditory improvements by performing multidimensional analyses of electrophysiological measurements, postoperative speech perception assessments and noise reduction technologies [36,37]. In this regard, there are methodological differences in the existing studies, the fact that the data sets are quite different from each other, and there are various limitations regarding the reliability of algorithms that have only recently been developed and used in the field of otolaryngology and audiology [10,38,39,40]. Considering recent research, it is evident that most of the work has focused on neurotology, and no review has specifically examined the research conducted in audiology. The aim of this systematic review was to evaluate and compare machine learning models used to predict auditory performance in cochlear implant users.

This study was conducted in accordance with the PRISMA [Preferred Reporting Items for Systematic Reviews and Meta-Analyses] guidelines and aimed to examine in detail the studies on cochlear implantation and machine learning related to audiological outcomes. It focused on the machine learning models used in the research, data types, parameters evaluated and their effects on decision processes in clinical applications. In this context, the aim was to compile the existing literature on individualized rehabilitation approaches for cochlear implant patients and identify potential areas for future research.

## 2. Materials and Methods

This study was written using the PRISMA 2020 guidelines. Although this study was conducted in accordance with PRISMA 2020 guidelines, the review protocol was not registered in a database.

This study comprehensively reviewed articles related explicitly to audiological outcomes of cochlear implantation and analyzed their results.

### 2.1. Data Resources and Search Strategy

The article screening process was carried out between September 2024 and January 2025. Studies conducted between 2010 and 2025 were included in the research. PubMed, Scopus, Web of Science, Google Scholar and IEEE databases were searched for studies. The keywords “Cochlear Implantation, Machine Learning, Hearing Loss, Auditory Performance After Cochlear Implantation, Electrophysiological Measurements, Speech Perception” were used in the searches.

### 2.2. Inclusion and Exclusion Criteria

Inclusion Criteria

- Cochlear implantation studies using machine learning models;
- International full-text studies published in peer-reviewed journals.

Exclusion Criteria

- Audiologic studies other than cochlear implantation using machine learning models;
- Theoretical studies;
- Abstracts published at conferences;
- Case reports.

### 2.3. Selection of Studies

Articles obtained as a result of literature searches from search engines were uploaded to the Rayyan AI software (https://help.rayyan.ai/hc/en-us/articles/4406419348369-What-is-the-version-of-Rayyan accessed on 3 April 2025). Rayyan is an application that uses AI-based algorithms to facilitate the systematic review process. In particular, it can predict article inclusion or exclusion decisions based on the referee’s previous selections using machine learning techniques. In addition, it is used to speed up the decision-making process by detecting duplicate or highly similar records through text similarity analysis. These AI-supported features aim to reduce the author’s workload, increase consistency, and improve the overall efficiency of study selection. Rayyan is a program that provides researchers with article screening, double-blind evaluation, categorization, and AI-supported recommendations. It can be preferred for time-saving and objective evaluation processes, as it allows the process to be followed in accordance with the PRISMA guide. In this study, Rayyan was used to speed up the literature review, clarify the inclusion and exclusion criteria, and make the research transparent by classifying studies by topic [41,42]. The filtering and tagging features of the program were used, and articles that met the inclusion and exclusion criteria were identified.

**Figure 1:**
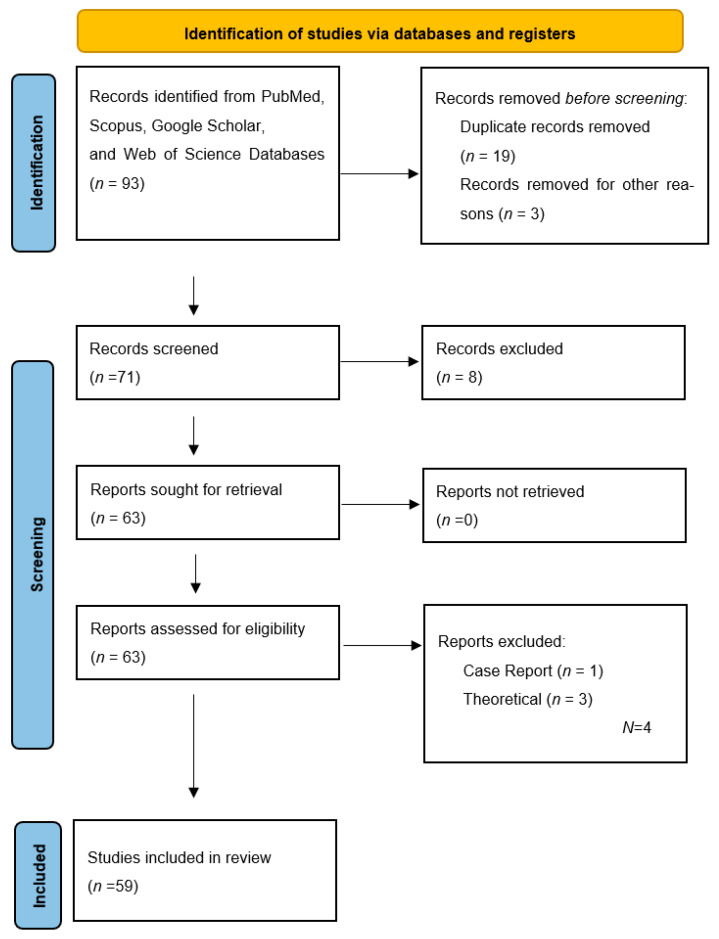
PRISMA Flow Date.

These tags were applied as “Machine Learning Modeling”, “Clinical practices in Adult Cochlear Implant”, “Clinical practices in Pediatric Cochlear Implant” and “Treatment and Rehabilitation Methods”. First, titles and abstracts were examined, and then, the full text was evaluated. A total of 93 articles were obtained from literature searches. Using Rayyan AI software, 34 duplicated and eliminated articles were identified and removed (Figure 1). While analyzing the data, the sample size, the machine learning model used, and the performance criteria were recorded. This systematic review analysed studies evaluating auditory performance in cochlear implant users. Since the studies were conducted in different fields, speech discrimination scores and signal-to-noise ratios and similar metrics were considered as success parameters as primary outcome variables. Secondary outcome variables include sensitivity, specificity and F1 score, which indicate the accuracy of machine learning models. All measures obtained from the studies were reviewed, and all auditory performance measures reported in the studies were evaluated. In this systematic review, comparisons between the groups identified in the studies (implant group–control group) were evaluated to assess the effectiveness of cochlear implantation and machine learning-based analyses. In this systematic review, variables such as participant age, gender, type of cochlear implant used and follow-up period were analysed but not evaluated because detailed information was not provided in all studies. Studies with missing data were not included in this study. In this review, the results of the individual studies are presented with summary tables including the intervention, participant characteristics and main results of each study. Sample sizes across studies were compared in pie charts, and general trends for the number of studies expected to emerge in this area over the years were summarised in line graphs. In this study, the articles were analysed by a single researcher. In this review, no specific methodology was used to assess reporting biases. The classifications made through the Rayyan application ensured that the screening process was carried out objectively.

### 2.4. Data Analysis

The machine learning models used in the articles and their success rates were analyzed comparatively. The data obtained are shown in tables using descriptive statistics. In this systematic review, findings from individual studies are described and combined. The results across studies were analysed by grouping them thematically, and similar findings were brought together. Instead of a statistical analysis, the results and methods applied in each study were compared, and general trends and main findings were summarised. In this process, the heterogeneity of the studies was taken into account. In the search process, a total of 93 records were obtained as a result of the searches in the database. Due to duplication and other reasons, 22 studies were excluded. After title and abstract screening, 4 studies that were identified as case reports and technical reports were excluded, and the remaining 59 studies were included in the full text review. The flow diagram is given in Table 1. Small sample sizes and limited dataset information were observed in some studies. However, due to the limited and new nature of the existing studies in the field, these studies were included in the review. It was observed that the included data were analyzed double-blind.

## 3. Results

The distribution and application areas of the articles analysed in the study are presented. The analysis of articles published between 2013 and 2025 shows that the use of machine learning techniques in cochlear implantation is increasing. With the widespread use of deep learning models since 2018, the number of studies in this field has increased. In the reviewed articles, machine learning models have achieved successful results in preoperative and postoperative candidacy evaluations, especially in the cleaning and enhancement of speech signals in noisy environments, and machine learning models developed specifically for cochlear implant users have achieved successful results in preoperative and postoperative candidacy evaluations. However, it has been observed that it is aimed to develop automatically optimised applications in accordance with the individual needs of the users [26,41,42,43,44,45].

Additionally, deep learning clinical based models were frequently used together with traditional methods. However, it has been observed that most of the studies have problems such as limited data sets and lack of clinical validation [40,46,47,48,49,50,51,52,53].

**Figure 2:**
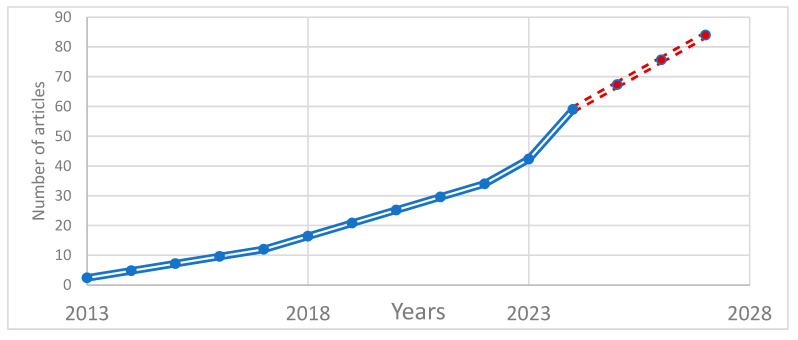
Changes in the number of articles by year. **Footnote:** Continuous lines indicate the current number of articles, while dashed lines indicate the number expected to reach in the future depending on the rate of increase.

In Table 1 and Figure 2, the distribution of the articles analysed in the study according to years and application areas is analysed. Sensitivity analysis, assessment of bias due to missing results, and analysis of the level of certainty of evidence were not performed in this review. However, considering the methodological diversity of the studies reviewed and the differences in the machine learning models used, care was taken to present the results as comprehensively as possible. The analysis of studies published between 2013 and 2025 shows a growing interest in the use of machine learning techniques in the field of audiology, particularly in cochlear implantation. As presented in Table 1, there has been a consistent increase in the number of publications over the years. During the 2013–2017 period, research mainly focused on early-stage machine learning, deep learning approaches and experimental data analysis. In 2018–2022, predictive model development and dataset optimization became more prominent, along with more frequent references to basic artificial intelligence applications. In the most recent period, 2023–2025, studies have highlighted integrating multiple machine learning methods, adopting innovative techniques and shifting toward broad-based deep learning applications using advanced algorithms.

This study presents a comparative analysis of the accuracy rates of machine learning applications utilized in research involving cochlear implant users in different sub-fields.

The success rates of the machine learning algorithms examined in this study are based on the model evaluation methods specified in the relevant literature. In these studies, it was observed that k-fold cross-validation (mostly 5- or 10-fold) was widely used to increase the generalizability of the model performance. In addition, L1/L2 regularization methods were used, especially in linear and Bayesian regression models. Among the performance metrics used, accuracy is the most common, and in some studies, measures such as F1-score, ROC-AUC and mean squared error (MSE) were also reported. The distribution of these methods according to sample studies is presented in Table 2. These evaluation methods support the reliability and generalizability of the success rates reported in the studies (see Table 3).

The machine learning models used in the studies included in the review show significant differences in data type, algorithm structure and targeted outputs. In particular, multidimensional data specific to cochlear implant users, such as speech perception test results, electrode placement information, impedance values and user performance evaluations, have been decisive in model selection. While artificial neural networks (ANNs) stand out in black data analysis, more explainable structures, such as support vector machines (SVMs) and decision trees, have generally been preferred in classification tasks. For example, in some studies, users have been classified as “successful/unsuccessful”, while some models have aimed to predict threshold values or satisfaction levels. Ensembling algorithms such as random forest have also been preferred due to their feature selection and advantages in error reduction. The findings are shown in Table 3.

**Figure 3:**
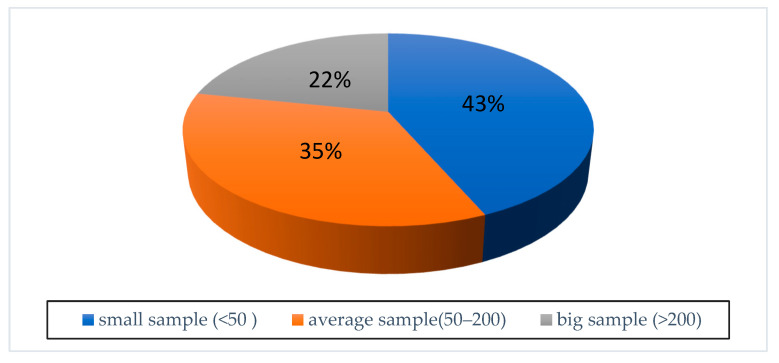
Data set characteristics.

The sample sizes used in studies on machine learning algorithms are an important factor for the generalizability and reliability of the methods. Depending on the problems considered, the datasets used and the topics, varying sample sizes have been identified. In this regard, the sample sizes of the papers included in this study are shown in Figure 3.

Machine learning has been used in the field of audiology and cochlear implants in different years with various approaches and applications. Initially, traditional machine learning methods such as basic decision trees and linear regression were often used, but over time, with the implementation of more complex algorithms and datasets, their use has expanded to EEG signals and biomedical approaches. The results are shown in Table 3.

In addition, Table 4 and Table 5 provides a detailed summary of the results of the reviewed articles. Each entry includes information on the machine learning model used, area of application, number of data points, number of participants, accuracy rate and an explanatory statement highlighting the study’s key findings.

The studies span from 2013 to 2024 and include various machine learning models such as support vector machines (SVMs), deep neural networks (DNNs), artificial neural networks (ANNs) and relevance vector machines (RVMs). The areas of application range from speech perception and speech in noise to electrode insertion depth and EEG optimization for cochlear implants. Accuracy rates vary across the studies, with some models achieving high success rates, such as 97.79% accuracy with SVM in speech intelligibility, and others report substantial improvements in noise reduction and speech discrimination.

## 4. Discussion

This study examined the distribution of machine learning applications in cochlear implant technology in the field of Audiology, application examples and accuracy percentages in different sub-fields, and the place of machine learning methods in clinical practice. When the sub-fields were examined, it was seen that the topics are mostly used in the applicability of automated machine learning methods to the effectiveness of intraoperative and postoperative tests in electrophysiological measurements, with the evaluation of models in pre-op candidacy predictions and speech understanding performance in noise. In addition, it is understood that evaluations have been made in a wide range of applications, from the use of deep learning-based speech recognition models using acoustic simulations instead of human subjects in speech perception evaluations to the development of emotion recognition skills from electroencephalography (EEG) data. Additionally, the effectiveness of cross-modal plasticity, machine learning applications in electrode design, and signal processing methods to enhance music perception in cochlear implant users are examined in detail. In this review, a total of 59 articles on machine learning techniques were analyzed. However, in the discussion section, specific papers were evaluated by excluding studies that were repetitive in terms of methodology, dataset, and performance metrics and findings. The selection of the articles in this section was based on criteria such as the quality of the datasets, the validation processes used and the reliability of the results. However, the discussion section is structured around specific themes to provide a comprehensive and in-depth analysis.

### 4.1. Preoperative Candidacy

In cochlear implant evaluations, preoperative assessment plays an important role in users’ postoperative performance and rehabilitation. Otolaryngologists and audiologists complete the candidacy process by evaluating parameters such as the degree and type of hearing loss, neuro-anatomical structure, age at surgery, residual hearing status, hearing aid use and receptive-expressive language age [57,58,59,60,61].

In a study of 587 individuals regarding the preoperative evaluation process, a andom Forest machine learning model including demographic and audiologic data was compared to a traditional guideline for the evaluation of individuals with hearing loss. The model was evaluated by cross-validation and reported high sensitivity (92%) and specificity (100%) using speech discrimination scores, patient age and hearing thresholds at specific frequencies. The specificity of the traditional method was reported to be 42%. The study shows that the traditional guideline may be inadequate for new indications, but the developed machine learning model may be more effective by providing individualized assessments [62].

In the context of preoperative candidacy assessment, Zeitler et al. (2024) [63] used a random forest machine learning model to predict the most relevant factors affecting postoperative residual hearing levels and determine implant candidacy in 175 cochlear implant users. The Receiver Operating Characteristic Curve (ROC) curve and Matheww Correlation Coefficient (MCC) measures were used to evaluate the performance of the study. Postoperative residual hearing thresholds and preoperative meningitis history were positively associated with preserving residual hearing, whereas sudden hearing loss, noise exposure and ear fullness symptoms were negatively associated. Accuracy measures were provided through ROC and MCC measures. These measures are used in machine learning and statistics to evaluate the performance of classification models. While the ROC measure can take values between zero and one, the MCC can take values between minus one and one. In this study, MCC values ranged between 0.38 and 0.52 and ROC values between 0.73 and 0.83. The study shows that machine learning successfully predicts acoustic hearing preservation [63].

Carlson M. et al. (2024) [64] developed a machine learning model to assist in selecting cochlear implant candidacy in adults with demographic data and behavioral audiometry measures in another study of 700 individuals. AzBio and Consonant–Nucleus–Consonant (CNC) sentence and word recognition tests were used for candidacy prediction. Random Forest, XGBoost (XGB), and Logistic Regression models were used to predict the results obtained from the tests, and a model that can predict cochlear implant candidacy was developed. In the study, it was stated that this model, which was developed with an 87% accuracy rate and 90% sensitivity, can be used in clinics [64].

As is known, there may be missing data in behavioral tests performed in implant candidacy due to limited time, patient test compliance, etc. A study evaluated the accuracy of prediction models to complete the missing audiometric data with 1304 audiograms whose pure tone audiometry data were complete at all frequencies. The performance of the estimation model was evaluated using cross-validation methods with various amounts of missing data and sparsity distributions. The results showed that the Multiple Imputation by Chained Equations (MICEs) machine learning model was able to estimate missing data up to six frequencies in an 11-frequency audiogram. In this study, the MICE machine learning model showed the best performance. More sophisticated machine learning methods (XGB, NN) performed better with more data but not as well as MICE. In this study, the researchers also reported that completing missing data increased the size of the dataset by 5.7 times compared with performing a complete analysis. With models similar to these models, it is stated that studies with more people can be conducted to prevent data loss [55].

When the success rates of these studies on preoperative candidacy assessment are evaluated, they are measured with different metrics. Despite the different measurement methods, it is seen that high success rates are achieved in candidacy prediction, but studies are limited. Although the application evaluated in preoperative evaluations is different, it is seen that the Random Forest method is often associated with high prediction rates.

### 4.2. Intraoperative–Postoperative Measurements

Cochlear implant surgery is a field with very comprehensive stages. In this situation, after the cochlear implant candidacy evaluation, electrode placement, and subsequent programming are critical regarding language development in children, quality of life and effective communication in adults. The most important criteria for successful results are cochlear implant application to the right patient, programming and follow-up [65,66,67,68,69,70,71,72,73].

There are different studies on machine learning in this field. In a study comparing patients programmed with the Fitting to Outcome eXpert (FOX2G) machine learning software method with patients programmed manually, 47 experienced cochlear implant users took part. The evaluations observed a 10 dB improvement in cochlear implant hearing thresholds at 6000 Hz and a 10% improvement in discrimination tests in patients programmed with FOX software. It was also reported that 89% of users preferred FOX programming to manual programming. This indicates that this software could be used for candidacy assessment [33].

Another study conducted with the artificial intelligence-based FOX algorithm compared the effectiveness of machine learning methods and traditional programming methods. Experienced clinicians with traditional methods first programmed fifty-five adult patients and then reprogrammed them with the FOX algorithm. Performance measurements were made using the CNC word and AzBio sentence tests. In the study, 84% of the patients showed the same performance with FOX programming. No significant difference in mean performance was reported between FOX and traditional methods. It was reported that the software used in this study may help less experienced clinicians to perform effective programming. Regarding patient satisfaction, 82% of the users were satisfied with the sound quality provided by FOX [38].

A study using machine learning models to predict electrode impedances after cochlear implantation in relation to postoperative measurements analyzed data from 80 pediatric patients using the Med-El Flex 28 electrode array to predict electrode impedances at 1, 3, 6 and 12 months post-op. Bayesian Linear Regression (BLR) and neural networks (NNs) gave the best results for most channels. In the study, patient age and intraoperative electrode impedance are important determinants in electrode impedance predictions. It is stated that different electrodes for electrode impedance can be better predicted with different machine learning algorithms. In the study, it was reported that the prediction accuracy of electrode impedance at the 12th postoperative month ranged between 83 and 100% [36].

Many variables affect the results after cochlear implantation. One of these variables is electrode placement and depth. Although electrode placement is evaluated with some electrophysiologic and imaging methods during surgery, unpredictable results may occur. For this purpose, Hafeez et al. (2021) used a robotic system for electrode placement in the cochlea. With this system, electrodes were automatically placed in the scala tympani. The electrode array was placed at a constant speed (0.08 mm/s) and three different angles (medial, middle and lateral), and the magnitude, phase, reactance and resistance components were measured. The effects of different placement routes were analyzed. One hundred thirty-seven electrode placement experiments were performed, and impedance data of eight electrode pairs were recorded in each experiment. Support vector machine and shallow neural networks achieved 86.1% accuracy with partial placement data. The study reported that machine learning methods can provide instant notification during electrode placement, which can help surgeons or robots place the electrode array more accurately [39].

In another study on electrode placement depth and electrode arrays, machine learning was used to predict the location of electrodes using telemetry results. The study included 118 patients, and lateral wall electrode arrays were placed in all patients, and the most successful model was Extremely Randomized Trees. The model successfully predicted the linear depth of electrode placement with an average error of 0.8 mm. Although accuracy rates were not reported in the article, the distance between electrodes was 2.1 mm. The study suggests that this method may be an alternative to computed tomography results, especially in children [74].

Postoperative speech perception results are very important in evaluating the success of cochlear implantation and monitoring the auditory performance of the user. These results can help to plan individualized rehabilitation programs. Changes in speech perception can be monitored and used to determine implant effectiveness and additional interventions when necessary. In a study using supervised machine learning techniques to predict postoperative speech perception results, the Hearing Noise Test results were evaluated using 282 variables (demographic, audiological) in 1604 adult patients. It was reported that the results obtained could be detected with 95.4% accuracy at the end of the post-op 12th month with neural networks (NNs) machine learning model prediction. It was reported that age at surgery, presence of tinnitus, vestibular function, and psychological status of the patient were factors affecting performance [10].

Skidmore et al. (2021) developed an objective prediction model using machine learning and electrophysiological measurements to assess the functionality of the cochlear nerve in cochlear implant users. Electrically Compound Action Potential (ECAP) measurements were used for objective assessments. In the study, an index indicating the state of the cochlear nerve was developed using machine learning methods such as linear regression and support vector machine. The study reported that the algorithms correctly classified patients with hypoplasic and intact nerves in the range of 91–95%. The given accuracy rates showed that machine learning algorithms could accurately discriminate between the two patient groups with ECAP data. This study also reported correlation coefficients ranging from 0.49 to 0.73 between cochlear nerve index and speech perception tests [75].

Preservation of residual hearing after cochlear implantation has become very important in recent years, and a study by Zeitler D. et al. investigated the use of machine learning techniques to predict residual hearing after cochlear implantation. Logistic Regression, support vector machines, and Random Forest Extreme Gradient Boosting (XGBoost) methods were compared. Retrospective data of 175 patients were used in this study. The patients’ pre-op and post-op results were evaluated. The XGBoost machine learning model showed the best performance, with 83%. Preoperative low-frequency hearing levels, history of meningitis and sudden hearing loss were reported to be significantly associated with residual hearing. In the study, especially tobacco use and abnormal anatomical structure were reported to affect hearing preservation negatively. The results of this study suggest that machine learning models can be used to create more realistic expectations for patients after surgery and to plan options such as Electroacoustic Simulation (EAS) [63].

Predicting the results after cochlear implantation in inner ear anomalies is important in determining which patients should receive cochlear implantation. Factors such as anatomical condition, auditory experience of the patient, etc., can significantly affect the results. Weng J et al. used machine learning methods to predict hearing and speech perception performance after cochlear implantation. In this study, the radiological features of the cochlea were examined using 3D segmentation, and a prediction model was developed using the k-Nearest Neighbors machine learning algorithm. This model provides 93.3% accuracy for hearing rehabilitation and 86.7% for speech rehabilitation. The study reported that cochlear volume and canal length are important determinants of post-cochlear implant performance [12].

In addition, cochlear implant success was evaluated in 70 children with hypoplasic nerves and normal cochleae after 2 years of follow-up. Hearing and speech development were evaluated using the categories of auditory performance (CAP), speech intelligence rating (SIR), meaningful auditory integration scale (MAIS), and meaningful use of speech scale (MUSS). The study determined the prediction accuracy of postoperative hearing and speech outcomes as 71% and 93%, respectively, using the support vector machines machine learning model. When the results were evaluated, it was reported that the cochlear nerve area and several nerve bundles were important factors for predicting cochlear implant outcomes. However, the study reported that age at implantation and residual hearing were not associated with cochlear implant outcomes. It has been reported that machine learning models can predict which patients may benefit more from cochlear implantation [14].

When the studies on intraoperative and postoperative measurements are evaluated, it is seen that machine learning techniques are used in subjects such as electrode placement depths, speech perception, preservation of residual hearing and prediction of cochlear nerve function. Different machine learning techniques have come to the forefront of studies. It is seen that the accuracy rates vary between 71 and 100% in the studies. Although the range seems to be exhaustive, most of the studies showed accuracy rates above 80%.

### 4.3. Speech Perception

With advancing technology, the question of how deep learning models can be used to evaluate the speech perception results of implants has been investigated. Speech perception can be measured as an objective reflection of users’ effective use of auditory performance. Speech perception tests help to optimize implant settings by reflecting how well sounds can be interpreted and discriminated. In addition, monitoring progression in the postoperative period and guiding the rehabilitation process is of great importance [76,77,78,79].

One study used the speech recognition algorithm of OpenAI’s Whisper Model. This model evaluates acoustic distortions and signal processing parameters such as the number of spectral bands, input frequency range and envelope cutoff frequency to simulate the auditory experience of implant users. The study reported that Whisper can be used to optimize implant signal processing parameters. In addition, it was reported that the application also exhibits human-like durability in noisy and quiet environments so that implant simulations can be evaluated effectively [47].

In another study, the speech intelligibility and signal-to-noise ratio performances of the model developed with machine learning to design and validate a digital signal processing plug-in for cochlear implants were evaluated. The study aimed to develop a new signal-processing plug-in to improve the signal-processing performance of cochlear implants. Wavelet neural network (WNN), a machine learning model, is used to create a plug-in for existing filter banks. The existing model is trained in particle swarm optimization. Success rates are evaluated in terms of speech intelligibility using the Short-Time Objective Intelligibility (STOI) metric. STOI is an objective method for evaluating speech intelligibility. It is especially used to measure performance in noisy environments. This metric takes a value between 0 and 1. If it is close to one, speech is considered intelligible, and if it is close to zero, speech intelligibility is considered low. In the study, speech intelligibility in STOI evaluations was measured as 0.834 in the WNN method and 0.762 and 0.813 in the DRNL and SPDN methods, respectively. In signal-to-noise ratio evaluations, it was reported that the WNN method obtained values of 2.470, DRNL, and SPDN methods −8189 and −1240 in STOI measurements. This new method is stated to perform better in terms of signal-to-noise ratio, speech intelligibility, and other performances compared with the existing digital signal processing techniques (DRNL and SPDN) in cochlear implants [49].

In an article evaluating post-op cochlear implantation outcomes, machine learning models used to assess word recognition performance at 12 months in 2489 post-lingual adult patients were compared. Artificial neural networks, Random Forests, XGBoost and gradient-boosting machine learning models were used. It is stated that the XGBoost model gives the best result. The absolute error rate of XgBoost was determined as 20.81. When data from different clinics were tested in the study, it was reported that the error rate was measured as a maximum of 16%. The main results of the study reported that increasing the amount of data did not increase the model’s accuracy. However, comprehensive and high-quality data affected the prediction accuracy of the model [13].

As is known, speech perception can be achieved by combining many factors. Pitch perception also plays an important role for cochlear implant users regarding music perception and speech discrimination. It is known that cochlear implants do not provide near-natural pitch perception due to limited spectral and temporal resolution, and new strategies are being developed for this purpose.

In a study evaluating pitch perception in cochlear implant users, a computational model was developed using spiking neural networks. This model was trained with digitally generated sounds and pitch cues to see how pitch perception changes in different environments. This model was used to compare a new model developed for cochlear implant users with the traditionally used ACE strategy. The study showed that the machine learning-based models performed better when the place and time cues matched. The results of this study suggest that in future cochlear implant designs, near-natural pitch perception can be achieved using machine learning models [48].

In another study using deep neural networks to estimate pitch perception, researchers developed a deep learning-based model to extract F0 fundamental frequency information from simulated cochlear implant signals. They investigated how cochlear implant signals carry pitch perception by varying the number of electrode channels and pulse rate. They reported that in quiet environments, the F0 accuracy of cochlear implants reached 90% when the number of channels increased above eight. The study reported that these results varied in noisy environments, and the accuracy rate was 75% with 20 channels and 2000 pulse rate, but when the number of channels decreased, this rate dropped below 50%. The study emphasized that deep neural networks can be effective in predicting F0, but the performance depends on parameters such as the number of electrode channels and pulses [56].

In a study where music was remixed using audio source separation algorithms to improve the music experience in cochlear implant users, Deep Convolutional Autoencoder (DCAE), deep recurrent neural network (DRNN), Multilayer Perceptron (MLP) and Non-Negative Matrix Factorization (NMF) methods were evaluated to separate vocals and instruments in songs. The performance of the algorithms was evaluated with metrics such as signal distortion ratio, signal-to-noise ratio and signal artifact ratio. Multilayer Perceptron was the most suitable algorithm for cochlear implant users due to user experience and low processing time [80].

In another study using deep learning-based sound separation technology to enhance the music listening experience of cochlear implant users, a Demucs neural network-based model was developed that separates music into vocal, rhythmic and harmonic accompaniment. The study used a pulsatile vocoder to simulate the hearing experience of cochlear implant users. Evaluations were made with the pulsatile vocoder on 15 individuals with normal hearing. A system that allows cochlear implant users to adjust instrument levels according to their subjective preferences has been developed. The proposed model succeeded more than objective and subjective systems in improving music perception for cochlear implant users. In particular, the personalized audio tuning feature significantly improved the user experience. It was reported that models requiring less computational power could be designed and integrated into cochlear implants in the future [81,82].

It has been observed that multiple machine-learning models have been compared in studies on speech perception evaluations. The applied machine learning methods varied, and the measurement results were evaluated using different metrics. Studies have been conducted on voice discrimination technologies, digital signal processing technologies, and pitch perception to improve speech intelligibility in cochlear implants. Machine learning techniques generally yield successful results in the studies.

### 4.4. Speech Perception in Noise

Understanding speech in noise can make communication difficult, even for individuals with normal hearing. This is a significant issue for cochlear implant users that complicates communication and the quality of daily life. Due to the limited frequency resolution and masking of the speech signal in cochlear implants, speech in noise can be challenging for cochlear implant users. Today, noise reduction technologies can give good results in constant noise. However, results may vary in fluctuating noisy environments [83,84,85,86,87].

Goehring et al. (2017) used machine learning methods to develop a speech enhancement algorithm based on neural networks to understand cochlear implant users in noisy environments better. The algorithm was applied to 14 cochlear implant users using three different noises: a speech-dominated noise, a noise that mimics the rhythm of speech and crowd noise. Two different algorithm training methods were created with and without speaker dependence. The study showed that the speech intelligibility of cochlear implant users improved by 5–6 dB in the noise that mimics the speech rhythm. However, the speaker-dependent algorithm performed better in all cases. The study suggests that although speaker-dependent models give better results, they need to be retrained for each speaker [32].

Cochlear implant users have difficulty understanding speech not only because of noise but also because of echo. Single and multi-microphone systems and integrated algorithms for the cochlear implant are used to reduce echo. However, the effectiveness of these systems is not one hundred percent in all conditions. Desmond J.M et al. developed a machine learning model to detect echo channel by channel. In the study, artificially generated echo models were evaluated using these algorithms. Machine learning models using Maximum A Posteriori and relevance vector machine were able to detect echo with about 90% accuracy. The study stated that the success rate of this model decreases when noise and echo are present together, but it can be improved with accurate user results [51].

Gaultier C. et al. (2024) also examined speech intelligibility in reverberant environments. They investigated how deep learning applications and the use of multiple microphones would affect the speech understanding of cochlear implant users. In the study, 12,000 different sound environments were simulated and modeled. The researchers tested single-microphone, three-microphone and multi-microphone deep learning-based algorithms. The study showed that using multiple microphones improved speech understanding by up to 10.3 dB. It is emphasized that algorithms should be standardized with different languages and speakers and that faster and lower-latency models should be developed [88].

In another study using a machine learning-based strategy to improve speech intelligibility in reverberation and noisy environments, reverberation is simulated using the Room Impulse Response (RIR) machine learning model. Accuracy rates were classified using the relevance vector machine learning model. Accuracy rates in the 60–80% range were reported for reverberant conditions and 30–60% for reverberant and noisy conditions [46].

In another study, to improve speech intelligibility for individuals with hearing loss, deep neural networks were used to analyze speech intelligibility for individuals with hearing loss. NC+DDAE, which consists of two main components, a noise classifier and a deep noise reduction autoencoder, was used in the study. While the developed model reached 19.1% of the control group under 5 dB SNR in two-speaker noise, this rate increased to 53% for cochlear implant users. In construction noise, while the control group was 45.4% under 5 dB SNR, this rate increased to 77% for cochlear implant users [40].

In a study aiming to improve speech intelligibility by recognizing environmental conditions in cochlear implant users, three features for environment detection were extracted from the ACE strategy, which is widely used in cochlear implant users, with the aim of enabling cochlear implants to classify environmental conditions automatically. Classification accuracies of the Gaussian Mixture Model, support vector machine model, and neural Networks models were reported to vary between 95 and 97%. The best results were obtained in both noisy and reverberant conditions. Despite limited training data, the support vector machine model was shown to be the most successful model, with a rate of 97.79% [50].

In another study on speech intelligibility, Kang Y. et al. (2021) evaluated deep learning-based speech enhancement algorithms that provide a trade-off between speech distortion and noise residual. This study identified a Long Short Term Memory (LSTM)-based speech enhancement algorithm. The system performance was tested using an envelope-based correlation metric. The study evaluated 10 normal hearing and 19 cochlear implant users. When the Wiener filter, which is considered a traditional method, and LSTM, which is a machine learning model, were compared, it was stated that LSTM performed better. It is stated that the proposed system can help cochlear implant users to understand speech better [54].

Recurrent neural network and SepFormer (Transformer-based advanced model) models were used to test speech intelligibility in 13 cochlear implant users with three different scales in another study investigating the effectiveness of deep learning-based machine learning methods in noisy environments. The SepFormer machine learning model was the most successful in speech intelligibility. Scores ranging from 74.7 to 78.2% were obtained in different noises. This study also evaluated the voice quality of cochlear implant users. In the evaluation method, where a maximum of 5 points can be obtained in the evaluation made with the mean opinion scores criterion, the SepFormer model received values ranging between 4.5 and 4.6 in different noises. This study shows that deep learning-based approaches successfully improve the speech intelligibility of cochlear implant users [34].

Developing a new model called Fused Deep ACE by combining the traditional ACE strategy with deep learning in bilateral cochlear implant users, the researchers aimed to facilitate the exchange of information between the two cochlear implants to improve speech understanding in noise and preserve binaural auditory cues. In this context, the models’ noise reduction, speech intelligibility, and speech quality performances were compared using speech and noise sounds. The best performance was obtained with the Fused Deep ACE bilateral ACE and deep ACE [41].

In another study using the Deep ACE model, DEEP ACE was the most successful in comparing the ACE model used with the traditional ACE Wiener filter and the models named TasNET+ACE for speech intelligibility in noise. In speech quality evaluations, the DEEP ACE model scored 91.8 in quiet and 75.3–79.6 in noisy environments on a 0–100 scale using the MUSHRA test. In speech discrimination tests, this rate was between 63.1 and 64.1%. The study shows that a deep learning-based voice coding strategy has excellent potential in cochlear implant users [42].

When the accuracy rates in studies examining speech intelligibility in noise are examined, it is seen that they vary between 30 and 97%. The varying rates indicate that different machine learning-based models should be developed, especially in reverberant environments, and tests should be performed in many noisy environments. However, despite the limited training data, it was observed that the support vector machine algorithm achieved the highest accuracy in noise comprehension test conditions.

### 4.5. Other Studies

Considering the problems experienced by cochlear implant users, it is known that they have problems with noise and emotional skills, such as emotion recognition. Due to the limited spectral and temporal resolution of cochlear implant technology, it is tough for cochlear implant users to understand musical perception and emotional speech expressions. Acoustic cues such as tone of voice, pitch changes, rhythm and timbre are the basic parameters of emotional perception. Since cochlear implant users cannot wholly and accurately encode these acoustic cues, they have difficulty distinguishing expressions such as surprised, sad, happy or neutral. This can result in difficulties in social adaptation and quality of life, including in patients with tinnitus symptoms [89].

In a study on this subject, the emotion recognition abilities of cochlear implant users were analyzed using machine learning models by analyzing EEG signals. In this study, 63.6% of the users were unilateral users, and 36.4% were bilateral users, and there were 22 users in total. A total of 24 audio and 24 musical expressions were tested with happy, sad and neutral emotion categories. In the study, EEG patterns specific to emotional activity were analyzed with the Random Forest machine learning model. It was reported that the model made 7.5% more successful predictions in the triple classification (happy, sad, neutral), 7.8%, 6.6% and 8.1% more successful predictions in the happy–sad, happy–neutral, and sad–neutral classifications, respectively. In the study, especially in the sad category (1.2–1.5%), model accuracy increased at rates ranging from 1.2 to 1.5%, and machine learning models reported promising results in cochlear implant users [90].

As is known, cochlear implant users’ rehabilitation outcomes vary according to demographic and audiologic factors. One of the reasons for these differences is the adaptation process required for cognitive processes to process and analyze electrical signals. EEG is an important tool for assessing plasticity and evoked potentials in cochlear implant users. As it is known, EEG evaluations cannot be performed in cochlear implant users due to electrical artifacts.

In this context, a study evaluating the effectiveness of machine learning techniques to remove cochlear-induced artifacts reported that it was detected with 95.4% accuracy in 66 pediatric cochlear implant users using a support vector machine. This study reported that artifact-detected EEG channels could be cleaned by the Ensemble Empirical Mode Decomposition method. As a result of this study, it was stated that artifacts can be removed from EEG data with machine learning models and can be used in rehabilitation methods [52].

In a study evaluating the prediction results using a machine learning model in auditory performance outcomes in cochlear implant users, 13 EEG data from three users were used pre-op and post-op at 3, 6, 12 and 18 months. Auditory, visual and tactile stimuli were used in the study. EEG data were used to investigate how neural data were activated in different cortex regions. Tactile features were found to be the best predictor, with a success rate of 98.8% in the success rates obtained using the support vector machine. Machine learning can be used to evaluate cochlear implant performance [53].

As is known, speech perception and quality of life outcomes show significant variability in cochlear implant users. This study used the K means clustering algorithm to evaluate speech perception and quality of life in 30 adult cochlear implant users. The participants’ performance was divided into three groups: high achievement group, at-risk group and highest achievement group. The results were evaluated using the Reliable Change Index. As a result of the modeling, it was reported that the best predictors of cochlear implant success were music education and cognitive capacity, while quality of life results were similar [91].

Unlike all these studies, in another study that examined the relationship between phenotyping based on psychological symptoms using machine learning and the presence of tinnitus in adult users of cochlear implants, 99 users were classified as having and not having tinnitus symptoms. The Gaussian Mixture Model machine learning model was used to determine the association of symptoms such as anxiety, depression and insomnia with tinnitus. With this model, subgroups were created from patient data. The relationship between the groups and tinnitus was analyzed with statistical tests. In the study, the rate of tinnitus was found to be lowest in patients with low symptom levels (39.1%), while the rate of tinnitus was higher in phenotypes with high anxiety and insomnia levels [80%]. This study reported that individualized treatments can be beneficial by creating psychological profiles with machine learning in cochlear implant users.

In the effectiveness of machine learning models, prediction accuracies were determined using Random Forest Regression, Histogram Gradient Boosting, and Elastic-Net regression models for hearing loss progression in Enlarged Vestibular Aqueduct (EVA) anomaly, a type of inner ear anomaly. Audiological, genetic and radiological data were analyzed. Incomplete Partition Type 2 and Endolymphatic sac signal heterogeneity were identified as the most important indicators of hearing loss progression. The study reported that machine learning models remained in similar ranges in prediction success (R2 = 0.26–27), while Elastic Net regression reached the highest accuracy with R2 = 0.32. In the study’s findings, it is reported that machine learning prediction models are still low in EVA, and early MR scans are important in these patients [92].

In another study by Nobel J et al. (2023), they developed a machine learning model to reduce the energy consumed by cochlear implants while providing nerve stimulation. The study was determined in two stages: developing an auditory neural model and determining energy-optimized waveforms. It was stated that the convolutional neural network (CNN) could mimic the real neural model with an accuracy of over 99% and increased the computation time by five times. At the same time, it was reported that this model consumes 8–45% less energy than conventional square waves. The study’s findings suggest that machine learning models can be used to increase energy efficiency and reduce the size of cochlear implants [43].

When the studies are evaluated, different areas related to machine learning for cochlear implant surgery in audiology have started to be studied in recent years. Energy optimization, tinnitus applications, emotional emotion recognition and artifact reduction, and inner ear anomalies have also been studied. In artifact reduction and evaluation of EEG data, it is seen that success rates of over 90% have been approached.

In addition to these diverse application areas of machine learning in cochlear implant surgery, a growing body of systematic reviews has emerged in recent years, aiming to synthesize the predictive use of machine learning models for auditory outcomes. When these review studies are evaluated, important thematic distinctions and methodological variabilities across the literature become evident.

When the review studies in the literature are examined, it is seen that Crowson et al. (2020) comprehensively evaluated various usage areas (signal processing, surgical support, etc.) of machine learning applications in cochlear implant (CI) processes but did not develop a specific focus on outcome prediction [9]. On the other hand, Mo et al. (2025) systematically reviewed studies on predicting auditory functional outcomes in CI users with ML models, but there was a significant diversity in terms of model types, predictor variables and validation methods [11]. Patro et al. (2024) developed automated guidance systems for the identification of CI candidates using machine learning, thus focusing on candidacy processes rather than direct auditory performance prediction [93]. Shafieibavani et al. (2021) evaluated the predictive power and generalizability of ML models on large multicenter datasets, but showed that the potential for clinical use of the models remained limited due to high error rates [13]. Zhang et al. (2024) analyzed the general trends of ML in the field of communication sciences and disorders with bibliometric methods, but did not provide an analysis specific to Cıs [94].

This systematic review presents a unique and narrow focus on auditory performance prediction in cochlear implant users, in contrast to the broader thematic studies in the existing literature. By minimizing the potential confounding effects of multidimensional variables that may influence cochlear implant outcomes, such as age, etiology, duration of implantation and comorbidities, the effectiveness of machine learning models in predicting auditory performance alone was analyzed with methodological rigor. Thanks to this focused approach, the clinical interpretability of the findings was enhanced, and directly applicable information was generated for model development and standardization processes. The study provides a conceptual framework for the specific use of machine learning algorithms in a complex clinical problem, such as auditory outcome prediction in cochlear implant users and provides a strategic roadmap that can guide future studies in terms of both model selection and dataset standardization. Accordingly, the present systematic review provides both a theoretical foundation and a practical guide for the development of machine learning-based clinical predictions in the field of cochlear implantation.

### 4.6. Evaluation in Terms of Clinical Practice

Although accuracy rates are frequently emphasized in the evaluation of machine learning models, this alone is insufficient to reflect a model’s clinical effectiveness. In addition to accuracy rates, it was observed that different metrics such as sensitivity, specificity, F1 score, area under the ROC curve (AUC), error rate, mean absolute error (MAE) and root mean square error (RMSE) were used in the studies examined in this review. In addition, some studies predicted performance through regression models instead of classification tasks and reported different metrics. However, many of these models did not directly target individual auditory performances of cochlear implant users; instead, they targeted indirect parameters such as preoperative candidate suitability, electrode placement optimization or complication risk prediction. In this context, not only the prediction accuracy of machine learning models but also how the predictions are made, which data are highlighted and the explainability of the model in decision-making processes are of critical importance for audiology practice.

The integration of machine learning models into clinical applications is affected not only by the model’s accuracy but also by the training and practical skills of users (doctors, clinical technicians, etc.) on how to use these results. In this context, supporting models with explainable artificial intelligence (XAI) features will allow healthcare professionals to understand the decisions made by the model and question them when necessary. In addition, it is of great importance for models to be able to make fast and reliable decisions for direct usability in clinical applications. However, difficulties such as data incompatibility, ethical issues, and legal obstacles that may be encountered in the clinical environment should also be taken into account for the development of these applications. Therefore, a multidisciplinary approach and long-term follow-up are required for the adoption of clinical decision support systems in the healthcare field. Explainable artificial intelligence (XAI) refers to the ability of the model to explain its decisions understandably and transparently, especially in healthcare and clinical applications. The main goal of XAI is to overcome the “black box” structure of deep learning and machine learning models and to provide healthcare professionals with information on how these models make predictions, which features are effective in what way, and which criteria are used to make decisions.

In many studies included in the review, machine learning-based models were tested only on experimental or retrospective data sets but were not systematically validated in clinical settings. This situation significantly limits the integration of these models into clinical practices and poses potential risks to patient safety. Lack of clinical validation directly affects the reliability and internal and external validity of the model, as well as ethical responsibilities in healthcare delivery. The high accuracy rates of machine learning algorithms on experimental data cannot be considered a sufficient indicator for integrating these models into clinical decision-making processes because there may be significant differences between the findings obtained in experimental settings and clinical practices in real-life conditions. These differences are shaped by many factors, such as the diversity of the patient population, variability in data quality, environmental factors and how healthcare professionals interpret the model output. Therefore, models must be tested prospectively in controlled and observational studies for experimental findings to be transformed into clinical validity. In this context, instead of focusing solely on algorithmic accuracy, it is important to conduct multicenter, long-term and application-based studies that evaluate the performance of models in clinical settings. Such studies allow us to assess whether the model works reliably in various patient groups under different clinical protocols and healthcare systems. In addition, issues such as how model outputs are interpreted by healthcare professionals and to what extent they can be integrated into clinical decision-making processes should be investigated. As a result, to strengthen the clinical validity of machine learning-based models and enable the sustainable use of these technologies in healthcare, the gap between experimental and clinical data needs to be systematically addressed. In future studies, it is important to focus on the technical adequacy of the model and clinical validation processes to make real progress in this field.

In this study, machine learning techniques used in cochlear implant surgery, especially in the field of audiology, were examined. Among the machine learning techniques used in the study, Random Forest (96.2%), Bayesian Linear Regression and Extreme Maching Learning have the highest accuracy rates, while Deep Denoisıng Autoencoder (DDAE) (46.8%), Sepformer Seperatıon Transformer (59.5%) and recurrent neural network (RNN) (59.5%) have the lowest accuracy rates. It was observed that deep learning methods such as ANNs, DNNs and LSTMs were used especially in complex methods such as speech intelligibility and speech understanding in noise. In machine learning techniques, methods such as Random Forest and Bayesian Linear Regression are thought to be resistant to high accuracy rates, diverse and high numbers of datasets, as well as overfitting (overlearning while training machine learning and obtaining false high accuracy answers). However, it is thought that the low accuracy rates may be due to the difficulties of machine learning methods when processing time series. This suggests that it may be more appropriate to evaluate deep learning methods used in complex skills, such as comprehension in noise, by subjecting them to longer training periods with larger, complex data sets. At the same time, the success criteria of the studies included in our study were evaluated using different metrics and different methodologies. Most of the studies in this review used similar machine learning models (e.g., decision trees and artificial neural networks), which allowed the overall results to be consistent. However, some studies achieved higher accuracy rates using more advanced models. These differences are related to the variety of data sets used and differences in model optimisation techniques.

Significant differences were observed between studies; some studies tested accuracy with smaller data sets, while others presented more reliable results based on larger and heterogeneous data sets. These differences had a significant impact on the generalisability of the model. Most studies used standard performance metrics such as accuracy and F1 score, which facilitated the comparison of results. However, some studies used only the accuracy metric, which may have led to ignoring class imbalances. This situation is thought to affect the generalizability of the results. In a significant number of the studies analyzed in this review, it is seen that machine learning models were developed with data obtained from a limited number of participants. This fundamental limitation directly affects the accuracy rates and the generalizability of the developed models for clinical applications. Small sample sizes may result from various reasons, such as the difficulty of ethics committee approval processes, restrictions on data confidentiality and security, difficulties in reaching cochlear implant users, and limited access to the sample, especially in studies on pediatric or rare groups.

Despite these limitations, it is noteworthy that in most existing studies, various methods are used to improve model performance. In particular, the preference for techniques such as cross-validation, data augmentation, oversampling and feature engineering reveals the efforts of researchers to obtain more reliable results when modeling with limited data. However, it should be noted that these techniques are insufficient to ensure the models’ generalizability.

Since the accuracy of models developed with small samples is often evaluated only on the training data, it remains unclear how the model will perform when applied to a different population or an independent data set. Therefore, in future studies, clinical validation that models are developed with multicenter and large datasets covering individuals with different socio-demographic characteristics is expected to provide more robust and generalizable results. In addition, replicating existing models in different datasets and testing their external validity is critical for the clinical validity of machine learning-based systems developed in the field.

In conclusion, although studies conducted with small sample sizes are an important start in the literature, there is a clear need for multicenter, large-scale and highly representative studies in order to make progress in this field. Such studies will facilitate the integration of the developed models into clinical decision support systems and increase their adaptability to different patient groups.

Using machine learning-based systems in cochlear implant users brings ethical responsibilities not limited to technical accuracy. First, most of the data on which these systems are trained is sensitive medical information; therefore, data anonymization, secure storage, and ethical approval processes are important. In addition, the transparency of the algorithms in the decision-making process is a separate topic of discussion. Since the interpretability of models such as artificial neural networks, which have a “black box” structure, is limited, integrating explainable artificial intelligence approaches is recommended so clinicians can understand algorithmic predictions and question them when necessary. Another important point is that using machine learning systems to support clinical decisions does not mean assigning the role of the final decision-maker to these systems. In this context, it should be emphasized that the final clinical responsibility should remain with healthcare professionals and that algorithmic biases should be recognized. When evaluated from all these perspectives, machine learning applications to be developed in the field of cochlear implants should be evaluated not only in terms of technical performance but also in terms of ethical responsibility. There are some limitations in the review process. Firstly, the articles were analyzed by a single researcher, which may have the potential to make some differences in the decision-making process.

## 5. Conclusions

Finally, the diversity of methodological approaches and data sets used across studies may have led to a specific heterogeneity in the results and may have created differences in the findings. In future studies, performance can be examined in more detail by making more standardized measurements with larger data sets. In future studies, how the results will be affected by the combination of different machine learning models can be discussed. This study examines machine learning applications in cochlear implant surgery in detail, focusing on Audiology. Random Forest and Bayesian Linear Regression models can be used more widely in clinical applications. However, further research is needed to use machine learning models in more complex data sets and fields.

It is known that cochlear implant candidates constitute a highly heterogeneous group in terms of age, hearing loss type, etiology, implantation time and accompanying health conditions. However, the datasets used in existing studies generally do not adequately represent this clinical diversity, which limits the generalizability of the developed machine-learning models and their performance in extreme cases. Integration of various technical approaches is suggested to overcome this problem. First, it may be helpful to use data augmentation methods, especially synthetic sample generation techniques such as SMOTE or ADASYN, to reduce the imbalance between classes. In addition, a more representative and general dataset can be created by integrating data from multicenter and different sources. In order to better manage heterogeneity, it is also important to separate the data into subgroups according to variables such as age group, etiology or cognitive status and train separate models for these groups. However, transfer learning and domain adaptation methods can improve model performance in smaller and rare groups. Domain adaptation enables the model to adapt to new areas by transferring information between different but similar datasets. In order to increase the explainability of the model, the features on which the model makes decisions should be made understandable with methods such as SHAP (Shapley Additive Explanations) and LIME (Local Interpretable Model-Agnostic Explanations). Finally, in order not to be limited to instantaneous assessments, it will be possible to predict the long-term auditory and cognitive outcomes of individuals more accurately by including longitudinal data in the model. Considering these techniques with a holistic approach will significantly increase both the accuracy and clinical applicability of machine learning models.

## Figures and Tables

**Table 1:** Distribution of machine learning applications in the field of audiology by year.

Year	Number of Articles	Featured Topics and Methods
2013–2017	12	Early machine learning applications Early deep learning approaches, experimental data analysis
2018–2022	22	Predictive models, dataset optimizations Increasing use of “machine learning”, basic AI applications
2023–2025	25	Integration of multiple machine learning methods, innovative techniques Transition to broad-based deep learning applications, advanced algorithms
Total	59	

**Table 2:** **A Classification of Artificial Intelligence Methods Based on Algorithm Type, Application Domain, and Evaluation Metrics.**

Algorithm	Using Field	Evaluation Methods	Regularization	Performance Metrics
**MAP**	Speech in Noise	10-fold CV	-	Accuracy
**RVM**	Speech in Noise	Train-Test Split	L2	Accuracy
**GMM**	Speech in Noise	5-fold CV	-	Accuracy
**SVM**	Post-Op Speech Perception	10-fold CV	L1/L2	Accuracy, F1-score
**ANN**	Electrode Design	10-fold CV	Dropout	Accuracy
**DNN**	Speech in Noise	Train-Test Split	Dropout, L2	Accuracy, MSE
**KNN**	Speech Perception	5-fold CV	-	Accuracy
**Random Forest**	Electrophysiological Measurements	10-fold CV	-	Accuracy, ROC-AUC
**LSTM**	Speech in Noise	10-fold CV	Dropout	Accuracy, F1-score
**CNN**	Electrode Placement	Train-Test Split	Batch Norm, Dropout	Accuracy
**DDAE**	Speech in Noise	5-fold CV	-	MSE, Accuracy
**MLP**	Signal Processing	Train-Test Split	Dropout	Accuracy
**SepFormer**	Speech in Noise	10-fold CV	-	Accuracy
**ELM**	Electrode Design	Train-Test Split	-	Accuracy
**Bayesian LR**	Electrode Impedance	Bayesian prior	-	MSE, Accuracy
**GBM**	Candidacy	10-fold CV	-	Accuracy, AUC
**RNN**	Speech in Noise	5-fold CV	Dropout	Accuracy

**Table 3:** Accuracy ratings of machine learning techniques based on the relevant fields of study.

Method	Accuracy Ratio Range	Field of Use
Decision trees	91–96%	Intra-Post-Op Measurement
Maksimum A Posteriori [MAP]	91.7–96.2%	Speech in Noise
Relevance Vector Machine [RVM]	91.7–96.2%	Speech in Noise
Gaussian Mixture Model [GMM]	95.13–97.79%	Speech in Noise
Support Vector Machine [SVM]	76–97.79%	Speech in Noise, Post-Op Speech Perception
Artificial Neural Networks [ANN]	65–89%	Electrode Design, Speech Perception
Deep Neural Networks [DNN]	18.2–44.4% [improved speech intelligibility]	Speech in Noise
K-Nearest Neighbors [KNN]	80.7–96.52%	Speech Perception-Quality of Life
Random Forest	73.3–96.2%	Electrophysiological Measurements,
Linear Regression	78.9–96.52%	Programming, Speech Detection
LSTM [Long Short-Term Memory]	71.1–82.9%	Speech in Noise
Convolutional Neural Networks [CNN]	54–99%	Electrode Placement, Speech Perception
Deep Denoising Autoencoder [DDAE]	46.8–77%	Speech in Noise
Multilayer Perceptron [MLP]	75–80%	Music Perception/Signal Processing
SepFormer [Separation Transformer]	59.5–74.7%	Speech in Noise
Extreme Learning Machine [ELM]	90–99%	Electrode Design
Bayesian Linear Regression	83–99%	Electrode Impedance Prediction
Gradient Boosting Machines [GBM]	87–93%	Preoperative Candidacy
Recurrent Neural Network [RNN]	59.5–74.7%	Speech in Noise

**Table 4:** Highlights and practices.

Period	Highlights and Practices	
	Machine Learning	Areas of Use in Audiology
Early Machine Learning Approaches	Use of basic machine learning methods such as basic decision trees and linear regression.	First experimental applications in the field of cochlear implant, especially post-op measurements
Early Deep Learning Approaches, Experimental Data Analyses	The introduction of more complex models, such as artificial neural networks (ANN) and support vector machines (SVM).	Early studies to improve cochlear implant performance by analysing experimental data.
Predictive Models, Data Set Optimizations	Use of predictive models (e.g., Random Forest, Gradient Boosting)	Prediction models in areas such as language development and speech perception after cochlear implantation.
	Data set optimizations and studies to improve model accuracy.	
Rising Use of ‘Machine Learning’, Basic Artificial Intelligence Applications	Expansion of machine learning methods in areas such as CI programming, electrode design and speech in noise.	Using basic AI applications (e.g., FOX system) to improve speech intelligibility of cochlear implant users
Increasing Emphasis on ‘Artificial Intelligence’, Model Comparisons	An increased emphasis on artificial intelligence (AI) and comparison of different models (SVM, ANN, Random Forest).	Using various AI models to predict hearing and speech performance after cochlear implant.
Integration of Multiple Machine Learning Methods, Innovative Techniques	Integrating multiple machine learning methods (e.g., LSTM, CNN, RNN).	Comprehension problems in noise with innovative techniques (e.g., deep learning-based noise reduction). Improving speech intelligibility of cochlear implant users.
The Transition to Comprehensive Deep Learning Approaches, Advanced Algorithms	Common use of deep learning models (e.g., Transformer, SepFormer) in CI.	Using deep learning models for the analysis of EEG signals and other biomedical data.
	Working with advanced algorithms (e.g., Multi-Task Learning, Deep ACE).	Improving cochlear implant users’ music perception and speech understanding in noise.

**Table 5:** Analysing the studies in different fields in detail.

	**Authors**	**Years**	**Machine Learning Model**	**Area of Use**	**Number of Data**	**Number of Participants**	**Accuracy Rate**	**Explanatory Statement**
1	Desmond, J.M. et al. [51]	2013	Maksimum A Posteriori (MAP), Relevance Vector Machine	Speech in Noise	simulation-		91.7–96.2%	Machine learning models were able to distinguish echo from other types of noise. The algorithms showed durability against different room and cochlear implant parameters.
2	Hazrati, O. et al. [50]	2014	Gaussian Mixture Model (GMM), support vector machine (SVM), Neural Network (NN)	Speech in Noise	720		95.13–97.79%	SVM model in speech intelligibility (showed the highest success with 97.79% accuracy rate).
3	Saeedi, N.E. et al. [48]	2017	Artificial neural network—ANN, Spiking Neural Network—SNN	Speech Perception	116	29	65–89%	Artificial neural network (ANN) has shown the best pitch ranking success when it uses spatial and temporal information together.Models using only spatial or temporal codes have lower performance.
4	Chu, K. et al. [46]	2018	Relevance Vector Machine (RVM)	Speech in Noise	-	15	10% improvement in reverberant environments, deterioration when noise and reverberation are combined	Partially successful machine learning applications
5	Lai, Y.H. et al. [40]	2018	Deep Learning/NC + DDAE (Noise Classifier + Deep Denoising Autoencoder)	Speech in Noise	320	9	Noise classification success rate 99.6% noise reduction: 67.3%	NC + DDAE gives at least 2 times, sometimes up to 4 times, better results compared to classical noise reduction methods
6	Hajiaghababa, F. et al. [49]	2018	Wavelet Neural Networks (WNNs), Infinite Impulse Response Filter Banks (IIR FBs), Dual Resonance Nonlinear (DRNL), Simple Dual Path Nonlinear (SDPN)	Speech Intelligibility	120		-	Wavelet Neural Networks (WNNs) showed the highest performance in both test and training sets.
7	Waltzman, S.B. & Kelsall, D.C. [38]	2020	FOX	Electrophysiological-Programming-Speech Perception		55	No statistically significant difference between manual programming and fox *p* = 0.65, and 0.47	With FOX, standardised rehabilitation, equal performance and improved patient experience have been found.
8	Kang, Y. et al. [54]	2021	LSTM	-	-	19	-	Deep learning based machine learning method for voice enhancement for speech understanding in noise
9	Hafeez, N. et al. [39]	2021	Support vector machine (SVM), Shallow Neural Network (SNN), k-Nearest Neighbors (KNN)	Electrode Insertion Depth-Intra Op	137		86.1–97.1%	Highly accurate classification of EA using different insertion measurements during the electrode array placement process
10	Gajecki, T. et al. [42]	2023	Deep Neural Networks—DNN, Deep ACE, Fully-Convolutional Time-Domain Audio Separation Network, Adam, Binary Cross-Entropy	Speech in Noise	-	8	SRT speech discrimination 63.1	The best model for noise reduction: Deep ACE
11	Pavelchek, C. et al. [55]	2023	Univariate Imputation (UI)Interpolation (INT), Multiple Imputation by Chained Equations (MICE), k-Nearest Neighbors (KNN), Gradient Boosted Trees (XGB), Neural Networks (NN)	Cochlear implant candidacy-Behavioural Tests	-	1304	93%	In real-world hearing tests, it has been shown that missing data can be safely filled in. In particular, RMSE = 7.83 dB was achieved, below the clinically significant error threshold of 10 dB.
12	de Nobel, J. et al. [43]	2023	Convolutional Neural Network (CNN), Evolutionary Algorithm (EA), Polynomial Elastic Net (PEN), Random Forest (RF), Gradient Boosting (GB), Multilayer Perceptron (MLP)	1,466,189 simulation samples, 12,441,600 different excitation waveforms			Accuracy—54–99%	The energy savings of these new waveforms may contribute to longer operation of CI devices with smaller batteries.
13	Zheng, Q. et al. [52]	2024	SVM—Support vector machine-EEMD-ICA	EEG-Optimisation	8448	91	95.44%	The SVM-based classification algorithm achieved 95.44% accuracy in automatically identifying channels containing cochlear implant artifacts.
14	Gajecki, T. & Nogueira, W. [41]	2024	Bilateral ACE, Bilateral Deep ACE, Fused Deep ACE	Speech in Noise	-	168	Speech intelligibility: 45–82%, noise reduction: 72–90%	Fused deep ACE model is the most successful model, 20–30% better speech understanding
15	Ashihara, T. et al. [56]	2024	Deep Neural Network (DNN)	Speech Perception	1024		-	Machine learning to improve speech perception in cochlear implant users

## Data Availability

The original contributions presented in this study are included in the article. Further inquiries can be directed to the corresponding author.
